# Reconstruction of complex knee wounds with a distally based gracilis flap and gastrocnemius myocutaneous flap: A case report

**DOI:** 10.3389/fsurg.2023.1109936

**Published:** 2023-02-08

**Authors:** Hyeokjae Kwon, Seokui Lee, Sunje Kim, Seung Han Song, Sang-Ha Oh, Joo-hak Kim, Hyunwoo Kyung, Ho Jik Yang, Yooseok Ha

**Affiliations:** ^1^ Department of Plastic and Reconstructive Surgery, Chungnam National University Hospital, Daejeon, South Korea; ^2^ Department of Plastic and Reconstructive Surgery, College of Medicine, Chungnam National University, Daejeon, South Korea; ^3^ Department of Plastic and Reconstructive Surgery, Chungnam National University Sejong Hospital, Sejong, South Korea

**Keywords:** case reports, plastic surgery procedures, surgical flaps, lower extremity, knee

## Abstract

A gastrocnemius muscle flap is a versatile option for covering the proximal one-third of the lower leg and around the knee. On the other hand, it is of limited use in patients with short gastrocnemius muscle or insufficient volume. The authors present a case in which a knee soft tissue defect occurred in a very thin patient and was reconstructed using a gastrocnemius myocutaneous flap and a distally based gracilis flap as a supplementary flap.

## Purpose

Skin and soft tissue defects around the knee are a challenge for reconstructive surgeons, and proper reconstruction is essential for patients to return to daily life (1). As the success rate of free tissue transfer increases, attempts to reconstruct knee defects using the procedure are increasing, but it is still difficult due to the deeply positioned recipient vessels (2). In general, knee defects can be covered by diverse muscle flaps, such as pedicled gastrocnemius muscle flap (3), distally pedicled gracilis flap (4), distal sartorius muscle flap (5), and distally based split vastus lateralis musculocutaneous flap (6). Among them, the medial gastrocnemius muscle flap is the “cornerstone” of the strategy for reconstructing defects around the knee and is considered to be a simple and safe method (7). However, the gastrocnemius muscle flap is often insufficient in volume and length, especially for the elderly with degenerated muscles or thin patients (8). The authors present a case in which a knee soft tissue defect occurred in a very thin patient and was reconstructed through a gastrocnemius myocutaneous flap and a distally based gracilis flap simultaneously.

## Case

An 80-year-old female patient with an underlying medical disease, hypertension, cerebral infarction, and heart failure was admitted to the hospital with a surgical site infection on the knee. She underwent total knee replacement arthroplasty four years ago to treat degenerative osteoarthritis. One year after surgery, a periprosthetic infection occurred, resulting in a soft tissue defect, and a free flap was performed to cover the defect.

The skin flap was fine, but the infection from the inside continued, causing soft tissue necrosis and dead space. Some openings on the skin occurred, with pus passing through it. The decision was to replace the artificial joint and capsule of knee reconstruction surgery after consulting with orthopedics.

**Figure 1:**
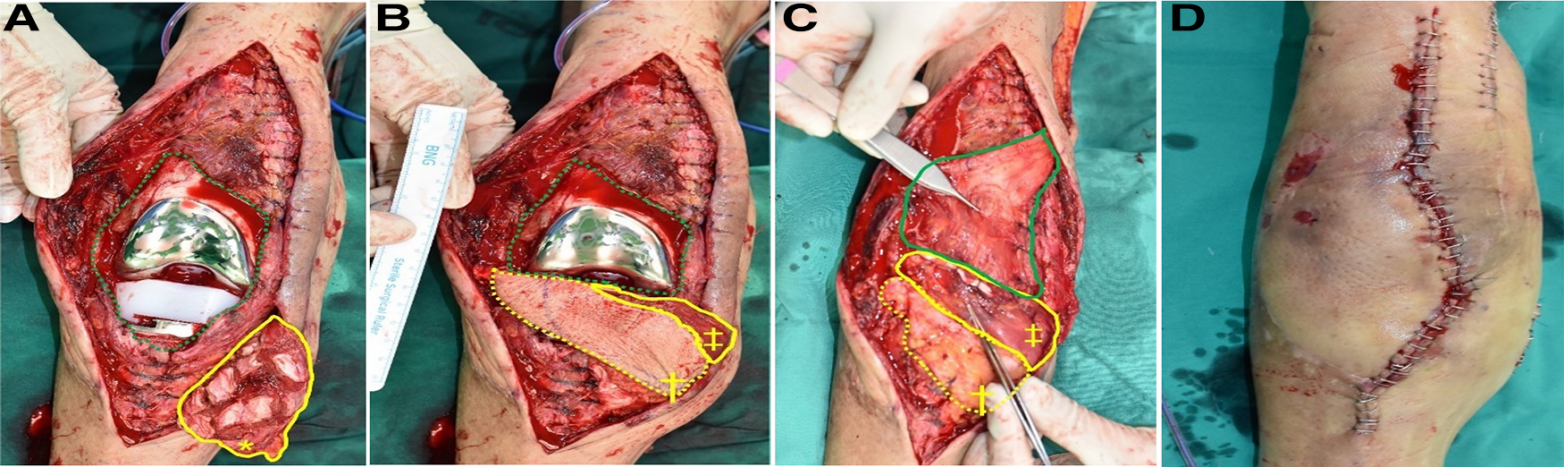
Intraoperative photographs. (**A**) 11 cm × 6 cm sized capsule defect (green dotted line), scored gastrocnemius muscle fascia (yellow bold line). (**B**) Gastrocnemius musculocutaneous flap covered the lower half. (**C**) Deepithelized skin island (yellow dotted line), the gastrocnemius muscle, and the distally based gracilis (green bold line) covered the defect was and sutured. (**D**) An immediate postoperative appearance after skin closure. (*, scored gastrocnemius muscle fascia; **†**, skin island overlying gastrocnemius muscle; **‡**, gastrocnemius muscle).

**Figure 2:**
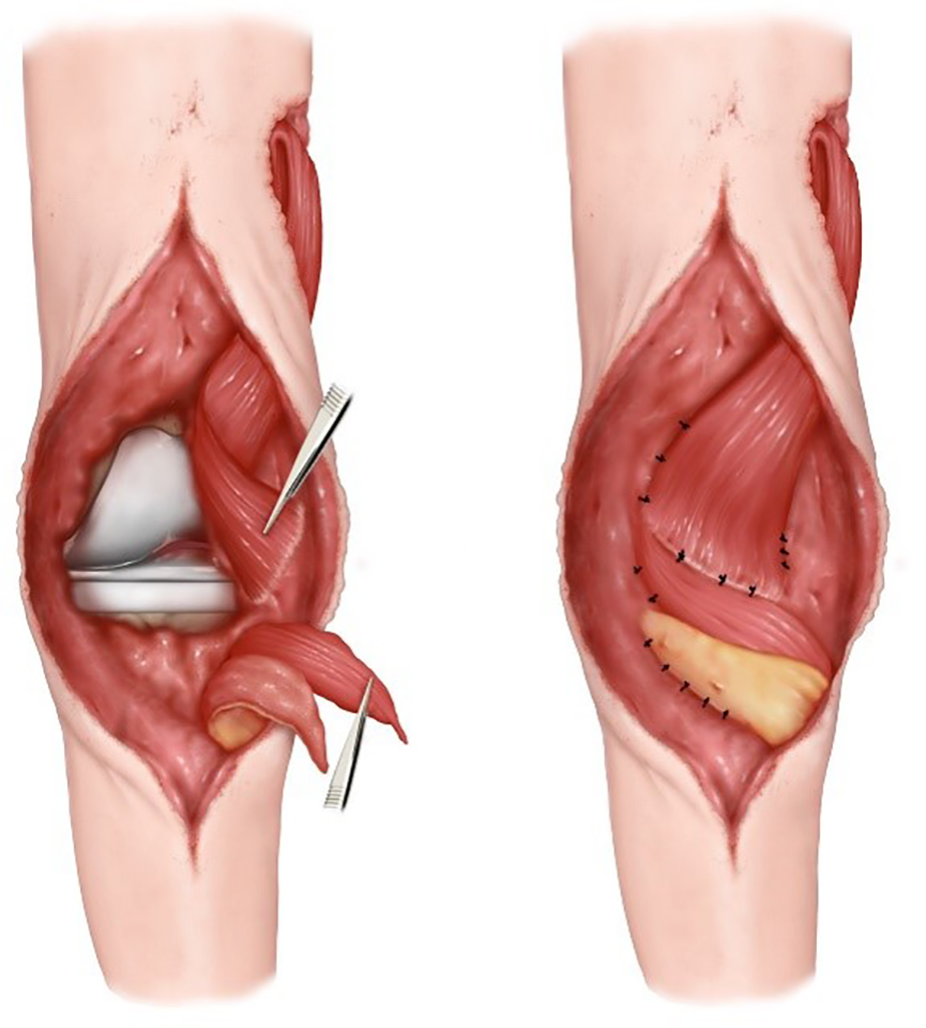
Schematic diagram.

The patient was very thin with a BMI of 15.81 (38 kg, 155 cm), and another free flap was recommended but declined due to previous surgical experience. Surgery for a capsule of knee reconstruction was decided with a gastrocnemius flap as the first choice. After replacing the artificial joint, the size of the capsule defect was confirmed as 11 cm × 6 cm. The capsule defect would be challenging to cover with only the gastrocnemius flap. Eventually, The flap volume was expected to be insufficient, so the following method was used (Figures 1, 2).

1. The gastrocnemius myocutaneous flap was elevated, and the distal halves were dissected and divided to cover the lower 50% defect. The skin island covering the gastrocnemius muscle was buried after deepithelization.
2. The gastrocnemius muscle fascia was scored to expand, and its origin was detached.
3. A distally based gracilis flap was used to cover the upper 50% defect.

The reconstruction operation took approximately two hours. The deepithelized skin and both muscle flaps showed bright red bleeding after the suture, indicating a healthy state. Primary closure of the skin envelope was possible owing to the previous free flap. The wound healing was uneventful and was well maintained two months after surgery.

## Discussion

The medial gastrocnemius muscle flap is considered a simple and safe method and is commonly used to reconstruct defects around the knee and proximal leg (9). Typically, the reach of the muscle is restricted to just below the knee, so the release of the medial femoral condyle and scoring of the deep fascia is used to increase the reach (10). In addition, the volume of the distal part of the muscle for defect coverage is small (11), and is even smaller when accompanied by disuse atrophy or aging, as in this case.

A chimeric gastrocnemius muscle and sural artery perforator flap were used to overcome this (12, 13). Because each component can be inset independently in a chimeric flap, it is highly preferable for 3-dimensional defects. On the other hand, the perforator needs to be mobilized carefully to prevent venous compromise, and according to its location, a sophisticated design suitable for the defect is required.

In this case, instead of completely separating the muscle component and the skin flap, only 1/3 of the distal area was separated and spread like a fan to increase the coverage area. As the patient had undergone a free flap before, she had relatively redundant skin. Hence, the skin flap covering the gastrocnemius muscle was used only to cover the capsule after deepithelization.

Even after covering with a deepithelized skin flap and gastrocnemius flap, a defect still occurred in the upper third; a distally pedicled gracilis flap was used to cover the rest (4, 14). A distally pedicled gracilis flap has been used for soft tissue defects around the knee, where a pedicled gastrocnemius muscle is inadequate or has already been used.

The gracilis muscle, based on the minor pedicles, would appear to run counter to the current appreciation of the vascular anatomy of this type II flap. In type II flaps, it is generally believed that such a flap cannot survive in its entirety on the minor vascular pedicle(s) unless delayed for 10–14 days (3). On the other hand, recent cadaveric and CT angiogram work has suggested that these minor pedicles may be sufficient to sustain the muscle without a delay procedure and was used successfully in three cases.

One of the most significant advantages of the gracilis flap, distally based on minor pedicles, is the short operative time. There are a few choices available to cover the superior medial defect of the knee. One of the choices is a free flap, but a gracilis flap takes a shorter operative time. Therefore, it is more suitable for elderly patients who are reluctant to receive a free flap.

A distally based gracilis flap has a locational advantage. If a gastrocnemius flap is insufficient to cover the defect, the remaining defect would be the upper part of the defect. The pivot point of the gracilis muscle is the superior medial part of the knee compared to the inferior pivot point of the gastrocnemius flap.

Finally, in the gastrocnemius flap, the volume of the distal part of the muscle for defect coverage is small because the muscle is cut near the tendon. On the other hand, in a distally based gracilis flap which was cut in the proximal muscular part. The end of this flap can be spread like fan, so can be used for wider defect. Therefore, the distal part of the flap is not narrow and can cover wide defects.

Despite the many advantages of a distally based gracilis flap, not many knee reconstructions have been performed using it. This is because a two-stage operation is considered necessary. Nevertheless, the technique should be considered by reconstructive surgeons or regarded as a useful supplementary flap that can be used when a gastrocnemius flap alone is insufficient.

## Conclusions

Many knee reconstructions are possible through the gastrocnemius muscle flap alone, but its role is limited in patients with insufficient volume. In this case, a free flap can be a better option, but there are cases where it is impossible. The author reconstructed the defect in a short time using the gastrocnemius muscle and skin island separately and using a supplementary flap in a patient who could not perform the free flap. The method shown in this case could be considered a way to reconstruct the knee in limited circumstances.

## Data Availability

The original contributions presented in the study are included in the article/Supplementary Material, further inquiries can be directed to the corresponding author.
